# Evaluation of the effectiveness of iodine prophylaxis in Poland based on the examination of school-age children in Opoczno

**DOI:** 10.1186/1756-6614-6-S2-A65

**Published:** 2013-04-05

**Authors:** Arkadiusz Zygmunt

**Affiliations:** 1 Polish Mother’s Memorial Hospital - Research Institute, Lodz, Poland; 2 Department of Endocrinology and Metabolic Diseases, Medical University of Lodz, Poland

## 

In 1997 a currently operating, obligatory model of iodine prophylaxis, based on mandatory iodization of household salt with 30 mg KI/kg, was introduced. Based on multiple surveys it was found that the model of prophylaxis employed in Poland has proved to be effective. Thus, Poland became a country with sufficient iodine supplementation on the population level. The prevention and control of iodine deficiency is a continuous process. It requires monitoring to be sustainable. There are many examples throughout the world where iodine deficiency has re-emerged as a public health problem, where once it was under control.

The aim of the study was to track the changes in iodine supply in recent years. School-age children were examined in 3 subsequent time points – in 1994, before establishment of iodine prophylaxis, in 1999 – 2 years after implementation of prophylaxis and in 2010 – 14 years after its implementation. 104 children (54 girls and 50 boys; age range from 6 to 15 years), 207 children (104 girls and 103 boys; age range from 7 to 15 years), and 174 children (94 girls and 80 boys; age range from 8 to 15 years) were examined in 1994, 1999 and 2010, respectively.

A significant increase in iodine excretion was observed, together with the decrease in goitre incidence and thyroid size. The median of UICs was 45.5 µg/l (1994), 101.1 µg/l (1999) and 100.6 µg/l (2010). Data demonstrate not only the effectiveness of iodine prophylaxis but also homogenous iodine intake in 2010 in comparison to year 1999, as the percentage of children in whom UIC was less than 50 µg/l decreased from 12.6 (1999) to 7.1% (2010).

It seems that at present the biggest problem is to define the reference values of thyroid volume, because values proposed by Zimmermann et al, which are currently being applied, still indicate the presence of goitre endemicity in Poland (15-18%).

Looking for a better tool to analyze the size of the thyroid gland, the results of thyroid volume (V) were compared to the body surface area (BSA) to give the ratio V/BSA. In our opinion, this ratio (V/BSA) better reflects changes in thyroid volume in particular time points and may be useful tool for comparing the size of the thyroid in different populations.

Fifteen years after the implementation of iodine prophylaxis in Poland the iodine intake has not only increased, but it is also more homogeneous. However, these values still oscillate around the lower values of normal range, which must be a cause for further concern as the widely recommended restriction of salt intake in everyday diet can lead to a decline in iodine intake below the recommended values.

